# Impact of a family medicine resident wellness curriculum: a feasibility study

**DOI:** 10.3402/meo.v21.30648

**Published:** 2016-06-08

**Authors:** Christine Runyan, Judith A. Savageau, Stacy Potts, Linda Weinreb

**Affiliations:** Department of Family Medicine and Community Health, University of Massachusetts Medical School, Worcester, MA, USA

**Keywords:** physician wellness, burnout prevention, residency education

## Abstract

**Background:**

Up to 60% of practicing physicians report symptoms of burnout, which often peak during residency. Residency is also a relevant time for habits of self-care and resiliency to be emphasized. A growing literature underscores the importance of this; however, evidence about effective burnout prevention curriculum during residency remains limited.

**Objectives:**

The purpose of this project is to evaluate the impact of a new, 1-month wellness curriculum for 12 second-year family medicine residents on burnout, empathy, stress, and self-compassion.

**Methods:**

The pilot program, introduced during a new rotation emphasizing competencies around leadership, focused on teaching skills to cultivate mindfulness and self-compassion in order to enhance empathy and reduce stress. Pre-assessments and 3-month follow-up assessments on measures of burnout, empathy, self-compassion, and perceived stress were collected to evaluate the impact of the curriculum. It was hypothesized that this curriculum would enhance empathy and self-compassion as well as reduce stress and burnout among family medicine residents.

**Results:**

Descriptive statistics revealed positive trends on the mean scores of all the measures, particularly the Mindfulness Scale of the Self-Compassion Inventory and the Jefferson Empathy Scale. However, the small sample size and lack of sufficient power to detect meaningful differences limited the use of inferential statistics.

**Conclusions:**

This feasibility study demonstrates how a residency wellness curriculum can be developed, implemented, and evaluated with promising results, including high participant satisfaction.

At a time when the need for primary care physicians in the US is only increasing, comparatively fewer medical school graduates are entering primary care specialties (1). High rates of burnout and relatively poor quality of life among primary care physicians are likely contributors (1–3), with as many as 60% of practicing physicians reporting symptoms of *burnout*, defined as emotional exhaustion, depersonalization (treating patients as objects), and a low sense of accomplishment (4, 5). As many as 68% of family physicians say they would choose a different specialty if possible (1). Physician burnout has been linked to reduced productivity (6) and poorer quality of care, including patient dissatisfaction, reduced adherence to treatment plans, increased medical errors, and decreased empathy (7–9).

Burnout is not contingent upon career stage nor is it correlated with years in practice (2, 3). In fact, evidence suggests residency may be the time when burnout is at its highest and wellness at its lowest as measured by exercise, sleep, seatbelt use, substance use, and overall wellness (4, 8, 10, 11). Residency training is difficult. The inherent expectations and demands of residency training in order to *become* a physician notably differ from the demands of *being* a physician, including lack of control of one's schedule, decreased and variable sleep schedules, as well as responsibility without authority. During residency, trainees are conscientiously gathering and learning to apply their medical knowledge. However, a ‘hidden curriculum’ about professionalism, coping strategies, and maintaining work/life balance parallels this formal training through culture and modeling by attending physicians. Work habits and patterns around self-care are developed and begin to concretize during residency training, creating a critical window of opportunity to enhance wellness and build resiliency to prevent burnout. In addition, with the implementation of the new accreditation system and the ACGME milestones for Family Medicine, achievement of specific competencies addressing wellness (e.g., Professionalism-4) is now explicit and expected.

Despite numerous reports of residency burnout, very few published trials of residency-based wellness curricula exist (12–16). The limited research has mostly focused on practicing physicians and emphasizes either mindfulness-based or cognitive-behavioral stress reduction (12, 17–22). The few studies conducted with residents don't address burnout *prevention* or offer clear evidence of effectiveness (12, 16) so the specific components or curricular model for an effective family medicine residency wellness curriculum remain unclear. This feasibility study contributes to a mounting imperative for residency wellness curricula that promote resiliency and teach skills to *prevent* burnout.

## Methods

The Worcester Family Medicine Residency (WFMR; 12-12-12 program) has three health centers serving socioeconomically diverse patient populations (urban, rural, and a Federally Qualified Health Center). All 12 PGY-II family medicine residents (9 females, 3 males) participate in a 4-week Physician as Leader (PAL) rotation. The PAL curriculum (Table 1) began in 2013 as a longitudinal experience that mirrors the IHI Triple Aim (23), including PAL I focusing on population health, PAL 2 on patient care, and PAL 3 on practice management. The wellness curriculum was embedded into the PAL 2 rotation for all residents.

**Table 1:** Wellness curriculum

**Goals**	
Understand the concept of mindfulness and how it relates to physician wellness and patient outcomes. Improve attention to the present moment and awareness of when it strays. Increase self-awareness (the capacity for self-observation and acceptance; cultivate awareness of one's own emotional temperature and resources to modulate as needed). Identify your values and explore how well your current actions are aligning with your personal values. Cultivate an attitude of curiosity and gratitude. Explore the use of narrative/appreciative inquiry methods. Develop personal tools for maintaining resiliency, preventing burnout (emotional exhaustion, depersonalization, and low sense of personal accomplishment) and enhancing empathic capacity.	
**Readings**	
**Required:** Curiosity, Fitzgerald, F. Annals of Internal Medicine 1999; 130: 70-72. Physician Resilience: What It Means, Why It Matters, and How to Promote It. Epstein, Ronald M. MD; Krasner, Michael S. MD Academic Medicine March 2013; 88(3): 301-303.	
**Suggested:** The Impact of a Program in Mindful Communication on Primary Care Physicians. Beckman, Howard B. MD; Wendland, Melissa; Mooney, Christopher MA; Krasner, Michael S. MD; Quill, Timothy E. MD; Suchman, Anthony L. MD; Epstein, Ronald M. MD. Academic Medicine June 2012; 87(6): 815-819. If Every Fifth Physician Is Affected by Burnout, What About the Other Four? Resilience Strategies of Experienced Physicians. Julika Zwack, PhD, and Jochen Schweitzer, PhD. Academic Medicine. March 2013; 88(3): 382-389.	
**Resources**	
Mindfulness/meditation apps and websites GPS for the Soul Donothingfor2minutes.com Someheadspace.com The Mindfulness App Insight Timer	
**Session objectives**	**Session activities**
Session I: Introduction to curriculum Burnout and mindfulness Self-assessments Mindful breathing and body scan Home practice	Brief centering exercise (Do nothing for 2 min) Review rationale and overall plan for curriculum Discuss the concepts of burnout and resiliency (TED talk video; didactic and discussion) Self-assessments with various measures: primary care provider stress checklist Introduction to mindfulness – savoring exercise with raisin Attributes of mindful practice: Attentive observation Critical curiosity Beginner's mind Presence Mindful breathing and body scan Ten breaths Drop anchor Notice five things SOLAR (Stop, Observe, Let it Be, and Return) Informal mindful practice in everyday living
Session II: Centering exercise and check in from last week Mindful communication – brief didactic overview Narrative medicine Cultivating gratitude	Centering breath exercise Three components of mindful communication: Mindfulness Narrative medicine Appreciative inquiry TED talk on happiness and gratitude Writing exercises on gratitude – three things grateful for Reflective writing with prompts Practicing listening skills: Reflective listening Generous listening Loving kindness meditation Home practice and writing/journal prompts
Session III: Centering exercise and check in from last week Exploring and defining our own intrinsic (professional and personal) values Provider bulls eye and retirement party worksheets Discussion	Breath as a central focus meditation or ‘leaves on a stream’ meditation You Tube video on work life balance and discussion Values clarification worksheet and discussion Provider bulls eye assessment with values defined Retirement party worksheet Walking meditation Home practice and journal writing prompts
Session IV: Centering exercise and check in from last week Self-care Letting go at the end of the day Developing boundaries Writing personal statement	Centering exercise Exploration of self-care activities and discussion Reflective writing on self-care and pair and share Strategies for letting go/boundaries Complete burnout prevention plan (Handout) Meditation on self-care and compassion
Follow-up: Monthly e-mails with mindfulness tips and reminders about using skills for 3 months after the curriculum ended	

The PAL curriculum was scheduled by rotation block, with a different rollout date for each health center, so it was done three times total. A behavioral science faculty member (CR) implemented the wellness curriculum every Friday afternoon for 2 h during the 4-week rotation with four residents per cycle. For 3 months following the curriculum, residents were sent monthly emails with reminders about implementing self-reflection skills, journaling, gratitude, and mindful breathing.

The study was a pre–post within subjects design, with a 3-month follow-up on the same set of four assessment measures. The pre-assessments occurred at the start of the PAL rotation for each site and the 3-month post-assessment occurred at three different times when PGY-IIs gathered for required didactics. Residents were informed of the confidential and non-evaluative nature of these matched pre–post assessments collected by a trained research assistant. Using paper/pencil administration, the measures included: 1) Maslach Burnout Inventory (4); 2) Self Compassion Scale (24); 3) Perceived Stress Scale (25); and 4) Jefferson Empathy Scale (26).

Statistical analysis was primarily descriptive. Paired *t*-tests were calculated to determine statistically significant changes. Because of the small sample size and non-normal data distributions, the non-parametric equivalent of the paired *t*-test was used. As results were identical, descriptive data from the paired *t*-tests are reported for ease of interpreting mean scores. A power analysis revealed insufficient power to find pre–post differences on all four measures so inferential statistics are not reported in the tables.

This project was granted exemption status by the University of Massachusetts Medical School's Institutional Review Board.

## Results

Of the 12 PGY-II residents, only 9 completed the pre measures while 12 completed the post measures. As shown in Table 2, residency wellness scores improved from baseline to the 3-month follow-up period. However, because of limited sample size and a resultant lack of sufficient power, few comparisons of the mean scores showed statistically significant improvements with the exception of the Mindfulness Subscale of the Self-Comparison Scale (SCI; *t*=−3.51; *p=*0.008).

**Table 2:** Pre- and post-measures of residency performance on standardized wellness scales^a^

Scale and subscales	Pre (mean, SD)	3-month post (mean, SD)
Self-Compassion Scale		
Self-kindness	5.67 (1.66)	6.56 (0.73)
Self-judgment	6.22 (2.17)	5.33 (0.87)
Common humanities	5.89 (1.36)	6.56 (1.88)
Isolation	6.11 (1.62)	6.67 (1.73)
Mindfulness	6.67 (1.73)	8.11 (1.69)
Over-identified	4.78 (2.17)	5.44 (1.51)
Overall self-compassion	35.33 (6.23)	38.67 (4.82)
Maslach Burnout Inventory		
Professional efficacy	24.78 (7.68)	26.89 (4.29)
Exhaustion	20.44 (9.36)	18.00 (9.88)
Cynicism	15.67 (8.94)	15.33 (8.07)
Perceived Stress Scale	18.11 (6.70)	14.78 (7.05)
Jefferson Empathy Scale	110.56 (18.30)	122.11 (5.49)

*N* = 9 PGY-II residents.

a Statistical testing was conducted between pre- and post-intervention measures but is not being reported due to the small sample size (and resultant lack of power) of residents participating in this pilot wellness curriculum implementation project.

Among other subscales, residents’ scores improved; that is, increases in reported self-kindness and compassion with concomitant decreases in reported self-judgment. Similar trends were seen in other measures; that is, residents reported less perceived stress at follow-up, improved efficacy, decreased exhaustion, as well as higher scores in empathy (which trended toward statistical significance). Interestingly, the range of scores on the Jefferson Empathy Scale shifted from pre- (71–130) to post- (115–134) measurement suggesting a more narrow range of scores toward increased empathy.

## Discussion

Reducing burnout among primary care providers is essential to achieving the IHI Triple Aim (23), but a paucity of effective programs to reduce or *prevent* burnout among residents has been evaluated (12). Preventing burnout may best be accomplished by building resiliency – a series of specific skills that, when cultivated, promote wellness. Encouraging resiliency skills during residency may be more effective than trying to undo patterns of thinking and behaving among practicing physicians when patterns are more concretized. This feasibility project demonstrates that a wellness curriculum can be executed within a residency with promising results and modest but dedicated faculty support. The small sample size resulted in lack of sufficient power to detect statistically significant differences pre/post-intervention; however, every measure trended in the desired direction and the SCI Mindfulness Subscale and the Jefferson Empathy Scale were both notably improved.

The strengths of this project include the use of validated instruments as well as development of a defined curriculum in modules that can be easily modified and implemented. The challenges of implementation include engendering leadership support, finding curriculum time, and engaging a well-trained faculty member. This specific curriculum required about 40 h of faculty time over the course of the year, including curriculum development time. The positive feedback from residents has led to a curriculum expansion, which is now embedded in *all* PAL rotations as well as monthly sessions throughout the year with PGYIIs and IIIs. This will permit an enhanced evaluation with follow-up. Further research in both curricular development and program evaluation is needed to determine whether introducing resiliency skills during residency impacts downstream outcomes such as burnout prevention, productivity, quality of care, expressed empathy, and patient satisfaction.

## Conclusion

Residency programs are increasingly implementing wellness initiatives to improve resident health, prevent burnout, and improve patient outcomes (27). The long-term impact of this wellness program is unknown; however, the positive subjective experience of residents and the corresponding culture shift to acknowledge, support, and promote longitudinal wellness activities within the WFMR program are meaningful and indubitable benefits.
